# Report Quality of Generalized Linear Mixed Models in Psychology: A Systematic Review

**DOI:** 10.3389/fpsyg.2021.666182

**Published:** 2021-04-22

**Authors:** Roser Bono, Rafael Alarcón, María J. Blanca

**Affiliations:** ^1^Department of Social Psychology and Quantitative Psychology, Faculty of Psychology, University of Barcelona, Barcelona, Spain; ^2^Institute of Neurosciences, University of Barcelona, Barcelona, Spain; ^3^Department of Psychobiology and Behavioral Sciences Methodology, Faculty of Psychology, University of Malaga, Málaga, Spain

**Keywords:** generalized linear mixed models, systematic review, empirical research, report quality, methodological review

## Abstract

Generalized linear mixed models (GLMMs) estimate fixed and random effects and are especially useful when the dependent variable is binary, ordinal, count or quantitative but not normally distributed. They are also useful when the dependent variable involves repeated measures, since GLMMs can model autocorrelation. This study aimed to determine how and how often GLMMs are used in psychology and to summarize how the information about them is presented in published articles. Our focus in this respect was mainly on frequentist models. In order to review studies applying GLMMs in psychology we searched the Web of Science for articles published over the period 2014–2018. A total of 316 empirical articles were selected for trend study from 2014 to 2018. We then conducted a systematic review of 118 GLMM analyses from 80 empirical articles indexed in Journal Citation Reports during 2018 in order to evaluate report quality. Results showed that the use of GLMMs increased over time and that 86.4% of articles were published in first- or second-quartile journals. Although GLMMs have, in recent years, been increasingly used in psychology, most of the important information about them was not stated in the majority of articles. Report quality needs to be improved in line with current recommendations for the use of GLMMs.

## Introduction

Data analysis strategies vary in complexity. The simplest techniques are those based on the general linear model, such as analysis of variance (ANOVA) or regression analysis, and these are the most commonly used in health and social sciences. These tests, for quantitative variables, require normally distributed data, homogeneity of variance, and independence of errors, assumptions that are not always satisfied in real-life settings. The generalized linear model (GLM) and the linear mixed model (LMM) provide a more advanced level of analysis. The GLM is a generalization of linear regression. The LMM allows for the inclusion of random effects factors and is useful when the assumptions of independence and constant variance are violated. Finally, at a higher level of complexity, the generalized linear mixed model (GLMM) includes random effects and generalizes the LMM to other types of response variables. The choice of model therefore depends on the metric of the response variable and its distribution, and on whether we are dealing solely with fixed effects or also random effects variables. In summary, each of the aforementioned models has different purposes and is suitable for specific types of data. In this article we focus on the use of GLMMs in psychology. For an in-depth discussion of GLMMs, readers may consult Dean and Nielsen (2007) and Stroup (2013).

The data obtained in health, social science and educational research often have skewness and kurtosis values that clearly deviate from the normal distribution (Micceri, 1989; Lei and Lomax, 2005; Bauer and Sterba, 2011; Blanca et al., 2013; Arnau et al., 2014; Bono et al., 2020). In fact, Micceri (1989) and Blanca et al. (2013) concluded that the distributions of real psychological data are usually non-normal. Micceri (1989) examined the distributional characteristics of 440 achievement and psychometric measures and identified several classes of non-normality. Blanca et al. (2013) analyzed 693 distributions reflecting cognitive ability and other psychological variables and found that most distributions were non-normal. In a more recent systematic review of empirical studies published between 2010 and 2015 in the fields of health, education, and social science, Bono et al. (2017) found a high percentage of non-normal distributions, the most widely used being gamma, negative binomial, multinomial, binomial, lognormal, and exponential, in that order.

It is clear from these studies that data in psychology frequently follow distributions other than the normal. This is important since although ANOVA has been shown to be robust to non-normality (Kanji, 1976; Khan and Rayner, 2003; Schmider et al., 2010; Ferreira et al., 2012; Blanca et al., 2017), it is not suitable for multinomial or ordinal data, and neither is it optimal with count data (Aiken et al., 2015). Consequently, applied researchers need to choose a statistical technique that is appropriate for their data, rather than seeking to find ways of using classical approaches at all costs (e.g., by transforming data so as to achieve normality or using non-parametric analyses). A further point to consider is that study designs that imply non-independence of observations due to nested sampling or repeated measures are common in psychology. Although applied researchers in psychology are aware that GLMMs are a flexible tool for analyzing such data, these models have rarely been used due to their complexity. In fact, various reviews of the analytic techniques most widely used in psychological research show that ANOVA is the most common (Edgington, 1964, 1974; Reis and Stiller, 1992; Schinka et al., 1997; Kieffer et al., 2001). More recently, Skidmore and Thompson (2010) and Counsell and Harlow (2017) found that ANOVA, correlations, and regression are the most frequently used techniques in psychology. A review by Blanca et al. (2018) of 663 data analysis procedures used in empirical studies in different areas of psychology and published in prominent journals during 2017 also found that ANOVA continues to be the most widely used technique, followed by regression.

Thiele and Markussen (2012) defined GLMMs as regression models that allow researchers to choose among various distributions and link functions in order to model a wide range of types of dependent variables through linear combinations of one or multiple predictor variables (fixed effects). The link function is a continuous function that transforms values of the response variable so that they match the scale of the linear predictors. This means there is a correspondence between distributions and link function. The first step of the modeling process is to find a suitable distribution and link function for the data at hand (Garson, 2013). For example, the natural distributions of count data are Poisson or, if the variance is larger than the mean (overdispersion), the negative binomial distribution; proportions and binary outcomes are binomial variables and the common link function is logit. However, although there is a natural link function for each distribution, there are also less commonly used alternatives that may suit the data better in some cases (Thiele and Markussen, 2012). For instance, binomial data may be modeled with probit link, while count data with large means may in some cases be modeled with identity link. The negative binomial distribution can also be modeled with identity link. Depending on the software package, other distributions and link functions may be available. Thiele and Markussen (2012) advised fitting models with different links to the data and using the link that yields the best model fit and parameter interpretation.

Another aspect to bear in mind is that GLMMs include random effects that can change when the study is replicated. Examples of random effects include subjects in a drug study, classrooms in an education study, or time in repeated measurements. In multilevel modeling, the intraclass correlation (ICC) can be calculated when using random effects. Hox (2002) describes the ICC as the amount of variance in outcomes that is explained by the grouping structure. The ICC can also be interpreted as the expected correlation between any two randomly chosen individuals in the same group (Hox, 2002). Heck et al. (2010, 2012) refer to the ICC as the proportion of total variance in the outcome due to within-unit differences at higher levels.

Overdispersion is another important issue to consider in relation to GLMMs. This phenomenon causes incorrect standard errors, which means that there is greater variance in the data that would be predicted or expected by the statistical model (Bell and Grunwald, 2011). In real data the variance often far exceeds the mean. Milanzi et al. (2012) found that Type I error rates were considerably inflated when overdispersion was ignored, implying that the probability of detecting a spurious effect increases. Overdispersion can therefore lead to erroneous conclusions in psychological research. An example from the field of addictive behavior would be when count results that are strongly skewed with many zero observations (e.g., total number of drinks, number of drinking problems or days of drinking) are combined with repeated assessments (e.g., longitudinal follow-up after intervention). In this context, Atkins et al. (2013) addressed the problem of overdispersion by analyzing longitudinal substance use data with hierarchical or multilevel models. Statistical models that assume normally distributed residuals applied to such data lead to incorrect confidence intervals and *p*-values. Additionally, when applying GLMMs, data with many zeros cannot be modeled by the corresponding probability distributions. Thus, GLMMs for count regression models, such as overdispersed Poisson, negative binomial regression, zero-inflated or Tobit are much more suitable for these types of data (Atkins and Gallop, 2007; Coxe et al., 2009; Hilbe, 2011; Thiele and Markussen, 2012; Aiken et al., 2015).

Although GLMMs are used to account for heterogeneity due to correlated measurements, Milanzi et al. (2012) concluded that there may be additional sources of heterogeneity that can affect statistical inferences if ignored. For example, heterogeneity may arise in longitudinal Poisson data, not only because of repeated measurements but also because of additional overdispersion.

It is also important to provide information about the estimation method. Without this information it is difficult to assess not only the suitability of the model used but also the reliability of the results obtained and the validity of interpretations. The choice of estimation method depends on the dependent variable and the random effects that are to be included in the model (Bolker et al., 2009). However, choosing the most suitable method from among the large number of possible options is not easy, and several aspects need to be considered. For example, the penalized quasi-likelihood (PQL) method yields biased parameter estimates if the standard deviations of the random effects are large, especially with binary data (Bolker et al., 2009). Generally speaking, PQL estimates are biased when the mean counts within groups are less than five for Poisson variables, or when the mean number of either successes or failures is less than five for binomial variables (Thiele and Markussen, 2012). Another example is that with binary data the Laplace approximation yields less biased estimates with a large number of clusters, whereas bias is greater with fewer clusters (McNeish, 2016).

Finally, attention needs to be paid to the information criterion, which is used to assess or compare different models and to consider the best model fit. The Akaike information criterion (AIC) is widely used for this purpose. The Bayesian or Schwarz information criterion (BIC) is very similar to AIC, but is less commonly used. The BIC tends to favor less complex models in comparison with AIC (Keselman et al., 1998). There are also variants of AIC (e.g., corrected AIC for small sample sizes, quasi-AIC for overdispersed data), as well as other information criterion alternatives. The choice here depends on the researcher’s judgment, as different indices may perform similarly well. Consequently, authors sometimes opt to calculate different goodness-of-fit indices in the same study.

Statistical software has contributed to the use of GLMMs. Although the theory and concepts underlying GLMMs began to emerge in the early 1990s, the incorporation of PROC GLIMMIX into SAS has made these techniques more accessible and applicable across the behavioral sciences (Charnigo et al., 2011). Other well-known software suites that include procedures for fitting GLMMs are R, STATA, and SPSS. Despite, however, the availability of this statistical software, these models remain complex. A further issue is that GLMM computation algorithms may fail to converge, most likely due to a complicated random and fixed effects structure.

Although GLMMs are gradually becoming more popular in psychology, they are still used less than in other disciplines, such as ecology (Bolker et al., 2009; Johnson et al., 2015; Kain et al., 2015), psychophysics (Moscatelli et al., 2012), biology (Thiele and Markussen, 2012), medicine (Cnnan et al., 1998; Platt et al., 1999; Skrondal and Rabe-Hesketh, 2003; Brown and Prescott, 2006), and, more recently, linguistics (Coupé, 2018; Elosua and De Boeck, 2020). In the field of clinical medicine, the use and quality of reporting of GLMMs was examined in a systematic review by Casals et al. (2014), who found that while these models became more widely used in the medical literature over the period 2000–2012, the report quality was poor. A systematic review of this kind has yet to be conducted in psychology, and in our view such a study would help to foster the correct application of GLMMs in the field.

The present study had two objectives. The first was to quantify how often GLMMs are used in studies published in prominent psychology journals and to analyze the trend in this respect. Second, and given the advisability of encouraging the use of these models and of explaining what information from the statistical analysis needs to be presented and how it should be interpreted, we conducted a systematic review to illustrate the use of GLMMs within psychology, focusing mainly on frequentist models. Our specific interest here was in report quality, and hence we examined whether articles published in the field of psychology provide all the information that is required when applying GLMMs.

## Methods

In order to address the first study objective we began by identifying all empirical studies in the area of psychology that were published between 2014 and 2018 and indexed in Journal Citation Reports (JCR). We then conducted a trend analysis of the use of GLMMs, taking into account the impact factors, quartiles, and categories according to JCR, countries of the journals, and countries of affiliation of the first author.

To fulfill the second study objective we examined the characteristics of studies and GLMMs described in empirical articles published in 2018 so as to analyze the information they provided. This was achieved through a systematic review conducted in accordance with PRISMA guidelines for the reporting of systematic reviews and meta-analyses (Moher et al., 2009). Supplementary Appendix 1 shows (in the right-hand column) our response to each of the items on the PRISMA checklist.

### Search for Information

The search was carried out in the Web of Science on July 15, 2019 and was refined to include only records in the area of psychology that were published in English during the period 2014–2018. The topics and initialisms considered all referred to GLMMs (i.e., generalized linear mixed model, hierarchical generalized linear model, multilevel generalized linear model, GLMM, and HGLM), and the specific search strategy was: “generali^∗^ linear mixed” OR “hierarchical generali^∗^ linear” OR “multilevel generali^∗^ linear” OR GLMM^∗^ OR HGLM^∗^. These topics could appear in the article title, abstract or key words.

### Selection of Studies

The selection of studies was performed independently by two reviewers (first and third author). In a first step, they screened the abstract of all records retrieved for the period 2014–2018, excluding duplicates, conference papers, book reviews, theoretical studies (statistical tests, new procedures, mathematical developments, comparison of models, etc.), tutorials or illustration of the use of analytic techniques, description of statistical software, systematic reviews, simulation studies testing models of analysis, and empirical studies in fields unrelated to psychology. Reports that served as marketing or advertising were also excluded, as were studies concerning the following: health service costs, drug or vaccine administration and their effect, patient medical variables without reference to psychological variables or mental disorders, diagnostic instruments, child care and pregnancy, nutrition and hygiene, demographics, and climate change. Of the 637 records originally retrieved, 312 were excluded (four duplicates, four conference papers, one book review, four theoretical studies, seven tutorials, two descriptions of software, four systematic reviews, 18 simulation studies, and 268 studies that did not pertain to the field of psychology). Of the remaining articles, a further nine were excluded as the corresponding journal was not indexed in the JCR in the year of publication. A total of 316 empirical articles published during the period 2014–2018 in JCR-listed journals and involving psychological variables (e.g., depression, anxiety, stress, aggression, skills, language, psychomotor development, etc.) were thus included. Inter-rater reliability for selection of articles was assessed with Cohen’s kappa (Cohen, 1968). The weighted kappa was 0.80, which can be interpreted as substantial agreement (Landis and Koch, 1977). The percentage agreement between the two raters was 90%. Disagreements were resolved by Discussion.

**FIGURE 1:**
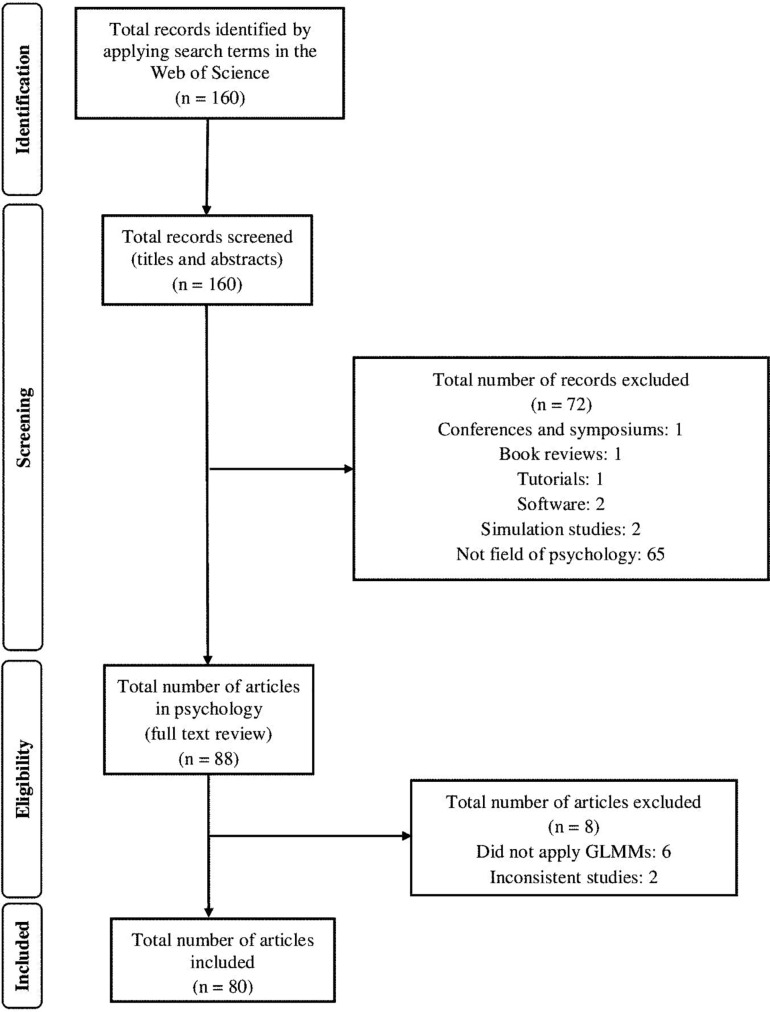
Flowchart showing the selection of articles published in 2018 and included in the systematic review.

In the next stage we examined the full text of the 88 empirical articles published in 2018. Articles were excluded if they only proposed applying GLMMs (e.g., study protocols) or if they contained inconsistencies or problems in the coding of variables. The agreement between raters in this second stage was 100% and a total of 80 articles published in 2018 were included in the systematic review. Figure 1 shows the process of selecting these studies.

### Data Extraction

To analyze the trend in the use of GLMMs over the period 2014–2018 we recorded the year of publication, journal title, impact factor, quartile and category according to JCR, country of the journal, and country of affiliation of the first author. The JCR category recorded for each journal was the one most closely related to psychology and with the highest rank, whether in Science Citation Index Expanded (SCIE) or Social Science Citation Index (SSCI).

For the systematic review of empirical articles published in 2018 we recorded the specific area of psychological research, characteristics of the design, sample, groups, and the dependent variables. Based on the classifications of Jiang (2007); Kauermann et al. (2010), Casals et al. (2014), and Tango (2017), we also looked for the important information about GLMMs that should be reported when applying these models (estimation method, link function, goodness-of-fit method, aspects related to the statistical inference of random and fixed effects, overdispersion evaluation, and statistical software). All this information was recorded for each distinct GLMM, that is, it was only recorded once in the event of identical models. For each empirical article we recorded the total number of GLMMs, whether or not they were identical, and how many were identical and how many different. Consequently, the number of GLMMs analyzed exceeds the number of articles reviewed.

Data for the systematic review were extracted independently by two reviewers (first and third author). Inter-rater reliability regarding variables in each article was 89%. Any discrepancy over the specific area of research, the study characteristics, and the characteristics of a GLMM was resolved by consensus among the two researchers, after first reviewing again the full text of the articles in question.

Finally, the frequency and percentages associated with the characteristics of the journals, studies, data, and GLMM analysis were calculated. This information provides an overview of change over time in the number of publications using GLMMs, journal productivity, and current practices in the use of GLMMs in psychology.

## Results

### Current Trends in the Use of GLMMs

**FIGURE 2:**
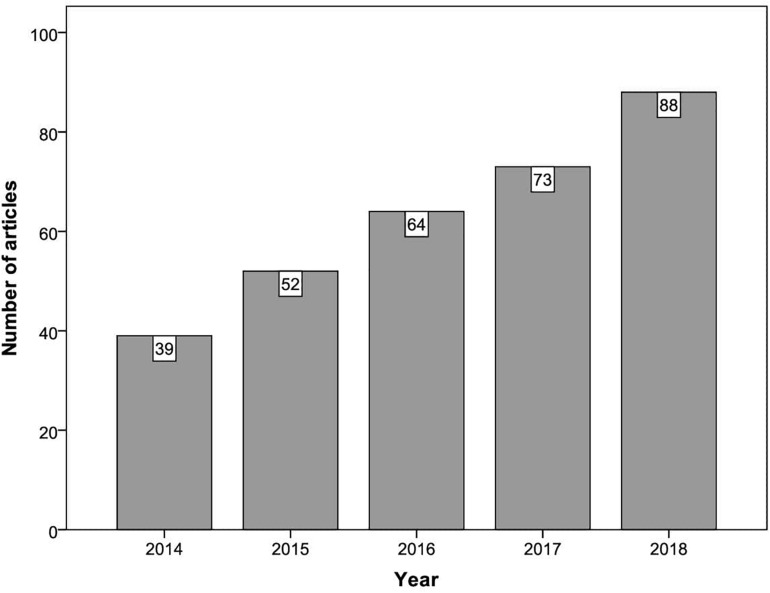
Number of articles by publication year.

Figure 2 shows how the 316 articles included in the first stage of analysis were distributed across the period of publication considered (2014–2018). It can be seen that there is a growing trend in the use of GLMMs in psychology, and that the number of empirical articles involving these models was more than twice as high in 2018 compared with 2014.

The journals that published the highest number of articles over the period 2014–2018 (see Supplementary Appendix 2) were: *PLOS ONE*, with 18 publications; *BMJ Open*, nine articles; *American Journal of Health Promotion* and *BMC Public Health*, six publications each; *Accident Analysis and Prevention*, *Addictive Behaviors*, *Drug and Alcohol Dependence*, and *Journal of Adolescent Health*, five publications each; *Addiction*, *AIDS and Behavior*, and *Annals of Epidemiology*, each with four publications; and *American Journal of Health Behavior*, *BMC Psychiatry*, *Consciousness and Cognition*, *Frontiers in Psychology*, *Human Movement Science*, *Journal of Clinical Psychiatry*, *Journal of Psychiatric Research*, *Journal of School Health*, *Nicotine and Tobacco Research*, and *Quality of Life Research*, with three publications each. The remaining journals had published one or two articles.

**FIGURE 3:**
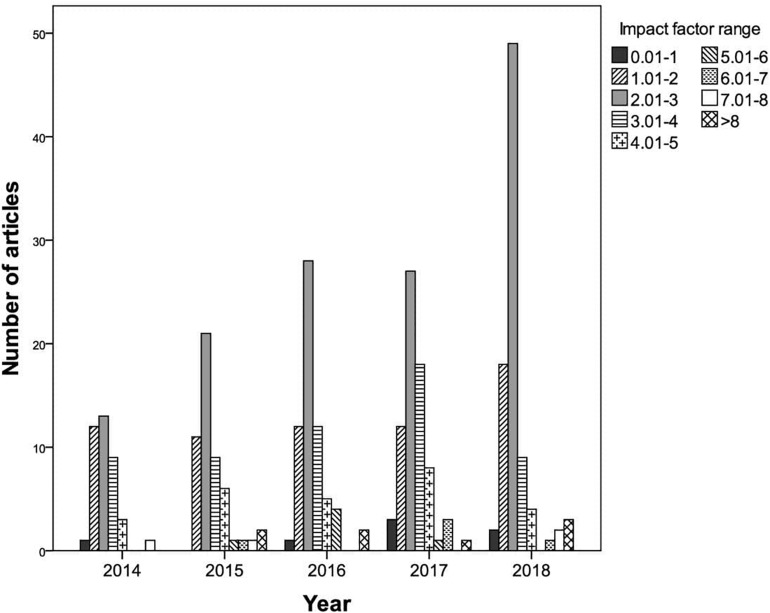
Number of articles in each impact factor range by publication year.

Regarding the impact factor of the journals in which they were published, seven publications ranged from 0.01 to 1 (2.2%), 65 from 1.01 to 2 (20.6%), 138 from 2.01 to 3 (43.7%), 57 from 3.01 to 4 (18%), 26 from 4.01 to 5 (8.2%), six from 5.01 to 6 (1.9%), five from 6.01 to 7 (1.6%), and four from 7.01 to 8 (1.3%). Eight publications exceeded an impact factor of 8 (2.5%). It can be seen in Figure 3 that for each of the years considered (2014–2018), the largest number of articles were published in journals in the impact factor range 2.01–3, with a notable spike in 2018 (55.7%).

**FIGURE 4:**
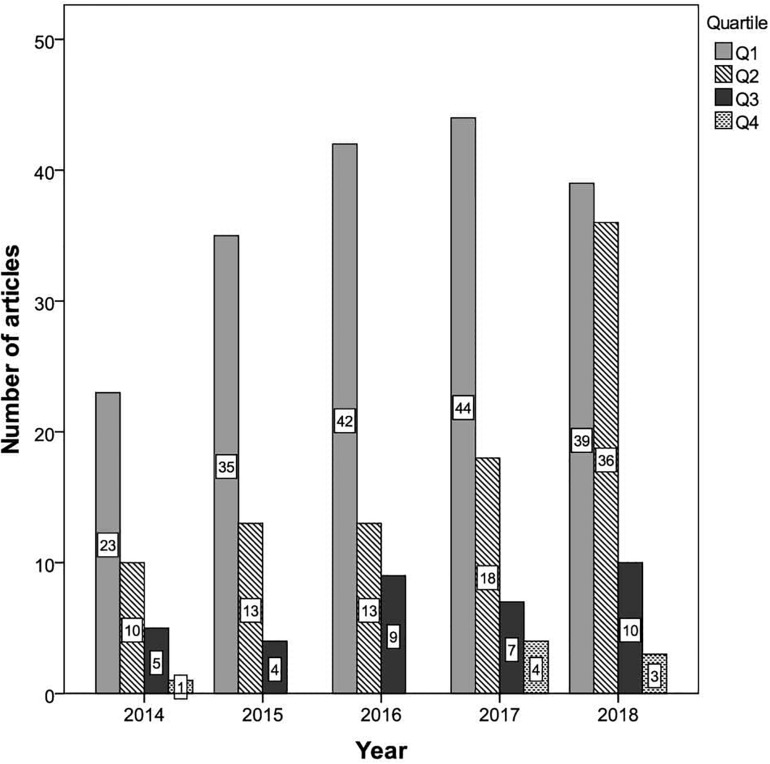
Number of articles by journal quartile and publication year.

With respect to the number of articles by quartile, 183 (57.9%) were published in first-quartile journals, 90 (28.5%) in second-quartile journals, 35 (11.1%) in third-quartile journals, and eight (2.5%) in fourth-quartile journals. Figure 4 shows the number of articles by journal quartile and publication year. It can be seen that in each of the years considered the highest number of articles involving GLMMs corresponded to first-quartile (Q1) journals, with a growing trend until 2017. Although Q1 journals still accounted for the highest number of articles in 2018, the number published in second-quartile (Q2) journals had also notably increased. Overall, 85.2% of the articles in 2018 that used GLMMs were published in first- or second-quartile journals. Across the 5-year period considered, only a small number of articles were published in third-quartile (Q3) journals, and there were hardly any in fourth-quartile (Q4) journals.

Regarding JCR categories (Supplementary Appendix 3), 43 articles were published in journals pertaining to *Public, Environmental, and Occupational Health*; 33 in the category *Substance Abuse*; 27 in *Psychiatry*; 20 in *Multidisciplinary Sciences*; 14 in *Medicine*, *General and Internal* and in *Psychology, Clinical*; 12 in *Psychology, Educational*; 10 in *Psychology, Multidisciplinary*; eight in *Gerontology* and in *Social Sciences, Biomedical*; seven in *Pediatrics*, in *Clinical Neurology*, and in *Psychology, Developmental*; six in *Psychology, Experimental* and in *Sport Sciences*; and five in *Ergonomics* and in *Psychology*. There were fewer than five publications in each of the remaining categories.

Productivity by journal country was much higher for the United States, with 172 publications (54.4%) over the period considered, followed by 87 articles published in the United Kingdom (27.5%), 20 in The Netherlands (6.3%), 15 in Switzerland (4.7%), five in Germany (1.6%), four in Australia (1.3%), three each in France and Japan (0.9%), two each in Denmark, Canada, and Ireland (0.6%), and one in Norway (0.3%).

Regarding the country of affiliation of the first author, the majority were from the United States, with 152 publications (48.1%), followed by 25 from Germany (7.9%), 22 from Australia (7%), 18 from the United Kingdom (5.7%), 16 from The Netherlands (5.1%), and 14 from Canada (4.4%). The remainder were from different countries around the world. By year, the highest number of articles involving GLMMs corresponded to 2018 and a first author based in the United States, with a total of 46 publications, compared with (for the same country affiliation) 25 articles in 2014, 26 in 2015, 20 in 2016, and 35 in 2017.

### Systematic Review of Studies Using GLMMs

We performed a systematic review of 80 empirical articles published in 2018. Studies using GLMMs were most frequently conducted in the areas of Clinical and Health Psychology (52.5%), Developmental, School, and Educational Psychology (13.8%), and Social Psychology (12.5%). Other areas identified were Environmental and Community Psychology (7.5%), Basic Psychology (5%), Neuropsychology (3.8%), Animal Psychology (2.5%), and Sport Psychology (2.5%).

Only 10 of the 80 articles reviewed (12.5%) presented Supplementary Information about the characteristics of the GLMMs. With respect to the number of GLMMs per article, 13 studies (16.3%) performed just one GLMM, 42 (52.5%) performed several identical GLMMs, and 25 (31.3%) conducted several GLMMs of distinct characteristics. The number of GLMMs performed in the same article ranged between one and 60, with two being the most common (26.3% of articles), followed by a single GLMM analysis (16.3%), three (13.8%), and four (11.3%). Overall, 23.9% of articles performed between five and 10 GLMM analyses, whereas only 9.1% conducted more than 10. The 80 articles reported a total of 464 GLMMs and the systematic review focused on the 118 GLMMs of distinct characteristics.

For each distinct GLMM we recorded characteristics of the design, sample, groups, and missing data (Table 1). The type of design was described as cross-sectional (39%), longitudinal or repeated measures (29.7%), or mixed (31.4%). In 39% of studies the authors manipulated the independent variables (in the remaining 61% they did not). Of the 118 GLMM analyses, the majority involved a sample of fewer than 500 participants (57.6%) and two groups (76.8%), although it should be noted that identifying the number of groups was not applicable in 41.5% of cases as the study analyzed the change in or association between variables (without a group comparison). As regards the number of repeated measures in longitudinal/repeated measures or mixed designs, the highest percentages corresponded to two (33.3%) and three (25%) such measures. Missing data was mentioned in 67.8% of the analyses. The GLMM is well-suited to longitudinal or repeated measures data because it appropriately handles missing data for response variables (Der and Everitt, 2006). However, only 14.2% of the studies that used GLMMs made some reference to missing data handling. Approximately half of the study designs (54.2%) had a hierarchical structure, and the majority of these (82.8%) involved two levels. A hierarchical structure was more common in cross-sectional designs (65.2% vs. 34.8% non-hierarchical) than in longitudinal/repeated measures designs (51.4% vs. 48.6%) or mixed designs (43.2% vs. 56.8%), although this association was not statistically significant, χ^2^(2) = 4.15, *p* = 0.126. Hierarchically structured data were usually nested in schools, hospitals, geographical areas, families, etc. The GLMM allows the analysis of data with a hierarchical structure through the inclusion of random effects in the model (Casals et al., 2014).

**TABLE 1:** Characteristics of the design, sample, groups, and missing data in the 118 GLMM analyses.

	*N* total	*N* (%)
**Type of design**	118	
Cross-sectional		46 (39)
Longitudinal/repeated measures		35 (29.7)
Mixed		37 (31.4)
**Manipulated independent variables**	118	
Yes		46 (39)
No		72 (61)
**Total sample size**	118	
Less than 100		34 (28.8)
101 – 500		34 (28.8)
501 – 1000		10 (8.5)
1001 – 5000		17 (14.4)
5001 – 10,000		8 (6.8)
More than 10,000		15 (12.7)
**Number of groups**	69	
2		53 (76.8)
3		5 (7.2)
4		10 (14.5)
6		1 (1.5)
Not applicable (no group comparison)	49	
**Number of repeated measures**	72	
2		24 (33.3)
3		18 (25)
4		8 (11.1)
5		6 (8.3)
6		4 (5.5)
7		1 (1.4)
12		2 (2.8)
14		1 (1.4)
20		1 (1.4)
45		1 (1.4)
70		2 (2.8)
Factorial		4 (5.5)
Not applicable (cross-sectional design)	46	
**Missing data reported**	118	
Yes		80 (67.8)
No		38 (32.2)
**Missing data handling reported**	118	
Yes		17 (14.2)
No		101 (85.6)
**Hierarchical structure**	118	
Yes		64 (54.2)
No		54 (45.8)
**Number of hierarchical levels**	64	
2		53 (82.8)
3		11 (17.2)
Not applicable (non-hierarchical structure)	54	

Table 2 shows the characteristics of dependent variables. Of the 118 GLMM analyses, 61.9% had a single dependent variable. In half of the analyses (50%) the measurement scales were nominal, while in approximately a third (36.4%) they were interval or ratio. The majority of nominal or ordinal scales (87.7%) had two response categories.

**TABLE 2:** Characteristics of dependent variables in the 118 GLMM analyses.

	*N* total	*N* (%)
**Number of dependent variables**	118	
1		73 (61.9)
2		16 (13.6)
3		4 (3.4)
4		6 (5.1)
5		2 (1.7)
6		5 (4.2)
8		2 (1.7)
9		3 (2.5)
10		3 (2.5)
12		1 (0.8)
13		1 (0.8)
14		1 (0.8)
31		1 (0.8)
**Measurement scale**	118	
Nominal		59 (50)
Ordinal		6 (5.1)
Interval/ratio		43 (36.4)
Count data		10 (8.5)
**Number of categories**	65	
2		57 (87.7)
3		2 (3.1)
4		3 (4.6)
5		2 (3.1)
6		1 (1.5)
Not applicable (interval/ratio or count data)	53	
**Distribution**	118	
Bernoulli		2 (1.7)
Binomial		23 (19.5)
Gamma		5 (4.2)
Multinomial		2 (1.7)
Negative binomial		3 (2.5)
Normal		7 (5.9)
Poisson		11 (9.3)
Non-normal		3 (2.5)
Not specified		62 (52.5)

Available statistical software can fit different response variables for distributions from the exponential family such as gamma, Poisson, binomial, negative binomial, multinomial, etc. The distribution of the response variable was reported in 47.3% of cases, the most frequent being the binomial (19.5%), followed by the Poisson (9.3%), the normal (5.9%), gamma (4.2%), and the negative binomial (2.5%). The lowest percentages corresponded to the Bernoulli and multinomial distributions (both 1.7%). In 2.5% of articles, reference was made to a non-normal distribution without specifying which. The distributions fitted in the GLMM analyses were the binomial and Bernoulli for nominal measurement scales with two response categories, the multinomial for ordinal scales with three or four categories, the Poisson and negative binomial for count data, and the normal, gamma, and Poisson for continuous measures scales (interval or ratio). These associations correspond to those cases in which the type of distribution was reported. The results indicate an association between the measurement scale and the distribution used, χ^2^(24) = 168.41, *p* < 0.001. The shape of the distribution was often not specified in those cases where the measurement scale was nominal or interval (55.9% of nominal scales and 58.1% of interval scales).

Table 3 shows the characteristics of the estimation and goodness-of-fit methods used in the GLMM analyses. Various methods of parameter estimation were described, namely the maximum likelihood (10.2%), restricted maximum likelihood (2.5%), Laplace approximation (1.7%), adaptive quadrature (0.8%), Gauss–Hermite quadrature (1.7%), and the adaptive Gauss–Hermite quadrature (0.8%). However, a very high percentage of analyses (82.2%) did not report this information, and hence other estimation methods may also have been used (e.g., penalized quasi-likelihood, pseudo-likelihood, etc.).

**TABLE 3:** Characteristics of estimation methods, link function, and goodness-of-fit methods used in the 118 GLMM analyses.

	*N* (%)
**Estimation method**	
Maximum likelihood (ML)	12 (10.2)
Restricted maximum likelihood (REML)	3 (2.5)
Laplace approximation	2 (1.7)
Adaptive quadrature	1 (0.8)
Gauss–Hermite quadrature	2 (1.7)
Adaptive Gauss–Hermite quadrature	1 (0.8)
Not specified	97 (82.2)
**Link function**	
Cumulative logit	2 (1.7)
Cumulative probit	1 (0.8)
Identity	4 (3.4)
Log	11 (9.3)
Logit/logistic	28 (23.7)
Not specified	72 (61)
**Goodness-of-fit method**	
Akaike information criterion (AIC)	10 (8.5)
Corrected AIC (AICc)	2 (1.7)
Watanabe AIC (WAIC)	1 (0.8)
Bayesian information criterion (BIC)	2 (1.7)
Deviance information criterion (DIC)	1 (0.8)
Log likelihood (LogLik)	2 (1.7)
–2 log likelihood (–2LL)	3 (2.5)
–2 restricted log pseudo-likelihood	1 (0.8)
Chi-square	2 (1.7)
AIC and –2LL	1 (0.8)
AIC and DIC	2 (1.7)
AIC, BIC, and –2LL	6 (5.1)
AIC, BIC, and LogLik	2 (1.7)
AIC, BIC, and chi-square	1 (0.8)
Not specified	82 (69.5)
***N* total**	118

Regarding the link function, this was not reported in 61% of the analyses. The link functions most commonly used were logit/logistic (23.7%) and log (9.3%). These percentages are associated with the distribution of the dependent variable, χ^2^(40) = 228.28, *p* < 0.001 (i.e., the logit/logistic link function is associated with binomial and Bernoulli distributions, the log link function with gamma and Poisson distributions, and the identity link function with the normal distribution). Fifty-two analyses reported neither the shape of the distribution nor the link function, 10 reported the link function but not the distribution, and 20 reported the distribution but not the link function (although in two of these cases it was merely stated that the distribution was non-normal). Only 36 GLMM analyses (30.5%) reported both the distribution and the link function. It should be noted that results from the GLMM using a normal distribution with the identity link function are equivalent to those obtained with the LMM.

Similarly, only 30.3% of the GLMM analyses reported the goodness-of-fit method. The most widely used was the AIC, either alone or in combination with other methods (18.6%). Variants of the AIC, such as corrected AIC for small sample sizes (AICc) and Watanabe AIC (WAIC), were also used (2.5%) as an improvement on the deviance information criterion (DIC) for Bayesian models (Watanabe, 2010; Vehtari et al., 2017). Other goodness-of-fit methods employed, either alone or in combination, were the BIC (9.3%), DIC (2.5%), log likelihood (3.4%), –2 log likelihood (8.4%), and chi-square (2.5%). Overall, 10.1% of the analyses combined two or three goodness-of-fit methods. The least used method was –2 restricted log pseudo-likelihood (0.8%).

Table 4 shows the inference of fixed effects in the 118 GLMM analyses. The purpose of statistical inference is to provide significance tests of the relationships between dependent and independent or predictor variables. The *p*-values refer to the existence of an effect, while the parameter estimates and confidence intervals provide information about the effect. Fixed effects hypotheses were most commonly assessed with the *t*-test or *F*-test (21.2%), and less frequently with the *Z*-test (5.9%), likelihood ratio test (5.1%), and chi-square test (2.5%). However, in the majority of cases (65.3%) the fixed effects test was not reported. The *p*-value was reported in 89.8% of analyses, the confidence interval in 50.8%, and the parameter estimation in 85.6% (50% coefficient estimate, 29.7% odds ratio, 3.4% incidence rate ratio, 1.7% risk ratio, and 0.8% relative risk ratio). More than half of the GLMMs (56.8%) included statistical modeling, that is, comparison of models by introducing or eliminating predictors. However, some analyses introduced all the predictor variables in a single step and do not compare different models (41.5%), or first chose the significant variables based on a previous study (1.7%). Finally, only 12.7% of studies reported the effect size.

**TABLE 4:** Statistical inference of fixed effects in the 118 GLMM analyses.

	*N* (%)
**Fixed effects test**	
*t*-test/*F*-test	25 (21.2)
*Z*-test	7 (5.9)
Chi-square test	3 (2.5)
Likelihood ratio test	6 (5.1)
Not specified	77 (65.3)
***p*-value reported**	
Yes	106 (89.8)
No	12 (10.2)
**Confidence interval reported**	
Yes	60 (50.8)
No	58 (49.2)
**Parameter estimation**	
Coefficient estimate	59 (50)
Odds ratio	35 (29.7)
Incidence rate ratio	4 (3.4)
Risk ratio	2 (1.7)
Relative risk ratio	1 (0.8)
Not specified	17 (14.4)
**Statistical modeling**	
Yes	67 (56.8)
No	49 (41.5)
First chose the significant variables	2 (1.7)
**Effect size reported**	
Yes	15 (12.7)
No	103 (87.3)
***N* total**	118

It can be seen in Table 5 that the random effects test was not usually reported (95.8%). Hypotheses concerning random effects variances were most commonly tested using the likelihood ratio test (3.4%). A single random effect was used in 56 GLMM analyses (47.5%), two random effects were used in 25 GLMM analyses (21.2%), and three or more in eight GLMM analyses (6.7%).

**TABLE 5:** Statistical inference of random effects and overdispersion evaluation in the 118 GLMM analyses.

	*N* total	*N* (%)
**Random effects test**	118	
Likelihood ratio test		4 (3.4)
*Z*-test		1 (0.8)
Not specified		113 (95.8)
**Number of random effects**	118	
1		56 (47.5)
2		25 (21.2)
3		6 (5.1)
4		1 (0.8)
9		1 (0.8)
Not specified		29 (24.6)
**ICC estimation reported**	118	
Yes		18 (15.3)
No		100 (84.7)
**Variance estimation reported**	118	
Yes		38 (32.2)
No		80 (67.8)
**Covariance structure**	72	
Autoregressive		2 (2.8)
Unstructured		7 (9.7)
Identity		1 (1.4)
Not specified		62 (86.1)
Not applicable (cross-sectional design)	46	
**Overdispersion evaluation reported**	118	
Yes		11 (9.3)
No		107 (90.7)
**Alternatives for handling overdispersion**	11	
Negative binomial		4 (36.4)
Overdispersed Poisson		2 (18.2)
Tobit		1 (9.1)
Not specified		4 (36.4)

*ICC, intra-class correlation.*

The intercept as random effects was reported in 47 GLMM analyses (39.8%). Random effects were usually the participant or subject (*n* = 47; 39.8%), such as patient, child, student, player, etc., and also the grouping variable (*n* = 44; 37.3%), such as classroom, community, hospital, school, geographical region, intervention group, etc. Ten GLMM analyses (8.5%) used the item as a random effect, and three (2.5%) used characteristics of subjects (e.g., age and gender). Some GLMM analyses used time as a random effect when data were collected repeatedly from the same individuals (*n* = 3; 2.5%). The interaction between variables was rarely used as a random effect (*n* = 4; 3.4%).

Table 5 also shows the percentage of analyses in which information was provided about the ICC estimation, the variance estimation, and the covariance structure. The ICC was reported in 18 GLMM analyses (15.3%) and the variance estimates of random effects in 38 analyses (32.2%). The covariance structure was only reported in 10 of the 72 analyses of data from longitudinal, repeated measures or mixed designs (13.9%). The unstructured covariance matrix was used in seven GLMM analyses, the autoregressive structure in two, and the identity covariance matrix in one.

Although it is important for GLMM analyses to assess and report the presence of overdispersion, it can be seen in Table 5 that only 9.3% of the analyses made reference to this. We found that four negative binomial models, two overdispersed Poisson models, and one Tobit model were used because data were overdispersed. These models were fitted to obtain standard errors corrected for the overdispersion parameter.

Finally, it can be seen in Table 6 that 85.5% of the analyses reported statistical software. The most widely used were SAS (36.4%) and R (31.4%), followed much less frequently by SPSS (6.8%), STATA (5.1%), HLM (3.4%), MATLAB (0.8%), Python (0.8%), and Mplus (0.8%). Among the analyses that used SAS, 32.6% made specific reference to the glimmix procedure, while 64.9% of analyses in R reported using the lme4 package. The specific package or procedure was not specified in 62.8% of SAS and 29.7% of R analyses.

**TABLE 6:** Statistical software and specific packages used in the 118 GLMM analyses.

	*N* software (%)	*N* package (%)
**SAS**	43 (36.4)	
Proc glimmix		14 (32.6)
Proc mixed		2 (4.6)
Not specified		27 (62.8)
**R**	37 (31.4)	
lme4		24 (64.9)
glmmADMB		1 (2.7)
Ordinal package		1 (2.7)
Not specified		11 (29.7)
**STATA**	6 (5.1)	
xtmixed		1 (16.7)
Not specified		5 (83.3)
**SPSS**	8 (6.8)	
Genlinmixed procedure		2 (25)
Not specified		6 (75)
**MATLAB**	1 (0.8)	
Fitglme		1 (100)
**Python**	1 (0.8)	
PYMC3		1 (100)
**HLM**	4 (3.4)	
Not specified		4 (100)
**Mplus**	1 (0.8)	
Not specified		1 (100)
**Not specified**	17 (14.4)	
*N* total	118	

## Discussion

Generalized linear mixed models combine linear mixed models (which incorporate random effects) and generalized linear models (that can handle non-normal data by using link functions and fitting distributions from the exponential family such as the binomial, multinomial, Poisson, gamma, lognormal or exponential). Thus, GLMMs offer a more flexible approach to analyzing data with distributions other than the normal, whether continuous or discrete. When using them, however, it is necessary to specify the distribution, the link function, and the structure of random effects. There are different methods for estimating the parameters and testing significance, and their suitability depends on the properties of the data.

Psychologists have much to gain from GLMMs because these models allow the analysis of categorical data and of count or proportional responses. By incorporating random effects, GLMMs also enable psychologists to generalize their conclusions. Despite these advantages, GLMMs rarely feature in research published in prominent psychology journals (Blanca et al., 2018). Consequently, the aim of the present study was to review the use of these models in psychology during the period 2014–2018. By conducting a systematic review of empirical studies published in 2018 that used GLMMs, we also provide more specific information about how these models are being performed and reported in psychology. This enabled us to identify gaps in the presentation of results and, therefore, to evaluate the quality of reports involving GLMMs in psychology. In doing so, we hope to foster the correct application of these statistical analyses and to encourage applied researchers in psychology to make greater use of them.

The number of articles using GLMMs in different areas of psychology increased over the period 2014–2018, although at a slower rate than was observed in clinical medicine by Casals et al. (2014) between 2000 and 2012. This is similar to what occurred with LMMs, employed prior to GLMMs, which were applied first in medicine and then more gradually in psychology (Bono et al., 2008). Our hope, therefore, is that GLMMs will also come to be widely used in psychology once researchers in this field become more aware of their advantages.

Our review of the period 2014–2018 included articles linked to the area of psychology from 198 JCR-indexed journals. The journal with the largest number of articles involving GLMMs was *PLOS ONE*. The large majority of studies using these models were published in first- or second-quartile journals, suggesting that the use of these more advanced analytical models is related to achieving publication in journals with a higher impact factor. As regards the JCR categories to which articles corresponded, the most common *were Public, Environmental, and Occupational Health*, *Substance Abuse*, *Psychiatry*, and *Multidisciplinary Sciences*. In terms of journal country, the majority of articles using GLMMs were published in the United States, followed by the United Kingdom. Regarding the country of affiliation of the first author, the majority were from the United States, followed (with much lower frequencies) by Germany, Australia, United Kingdom, Netherlands, and Canada.

Although gamma and negative binomial distributions are commonly used in health, education, and social sciences (Bono et al., 2017), our results suggest that they are still not sufficiently used. Consistent with what Casals et al. (2014) observed in clinical medicine, we found that the distribution of the response variable was most commonly binomial. Although the reported percentages are not entirely equivalent across the two studies [Casals et al. (2014) considered the number of articles, whereas we considered the total number of different GLMM analyses], there are consistent findings with regard to the type of distribution and other variables analyzed.

More than half of the GLMM analyses we reviewed did not report the shape of the distribution or the link function. Although there is a natural association between these two variables (Garson, 2013), this information needs to be reported as different link functions may be suitable for a given distribution. Furthermore, in the event of overdispersion it is usual to use an alternative distribution to the one fitting the data. Poisson data, for example, frequently present overdispersion.

It is also important to report the estimation method used in a study because it can influence the validity of GLMM estimates. The estimation method for each model was reported in only 17.7% of the analyses we reviewed, a percentage very similar to that found by Casals et al. (2014) in clinical medicine. Although, when the estimation method is not reported, it is likely that the authors used the default method (e.g., pseudo-likelihood is the default method in the glimmix procedure of SAS), this should nonetheless be specified.

In the field of psychology we found that the method most frequently used for parameter estimation was maximum likelihood. Although maximum likelihood underestimates the standard deviations of random effects, except in very large data sets, it is more useful for comparing models with different fixed effects (Bolker et al., 2009). In our review we found that maximum likelihood was mostly used with small samples (less than 100), although the corresponding aim was to compare models. Another alternative used was the Gauss–Hermite quadrature, which can produce more accurate estimates of the fixed effects and variance components in GLMMs (Pan and Thompson, 2003), although it is not feasible for analyses with more than two or three random effects (Bolker et al., 2009). In the two GLMM analyses where it was used, there was just one random effect and hence it was an appropriate method. We also found three analyses that used restricted maximum likelihood (REML). Although this method may be used, it is most suitable when the dependent variable can be modeled with a normal distribution (Thiele and Markussen, 2012). Of the three analyses that used REML, one was modeled with a binomial distribution, while in the other two the distribution was not specified.

Casals et al. (2014) found that the most common estimation method in clinical medicine was PQL, which is widely used as it is computationally fast (Breslow and Clayton, 1993). In the present review focusing on the field of psychology, only a small percentage of GLMM analyses reported the estimation method, and none of them used PQL.

The percentage of studies in the field of psychology that reported the goodness-of-fit method was lower than that observed by Casals et al. (2014) in clinical medicine (69.5% vs. 84.3%), although both these percentages are high. In both our study and that of Casals et al. (2014) the most widely used method (with similar percentages) was the AIC. Reporting this information is important for determining whether the method used is the most suitable. For example, seven of the GLMM analyses we reviewed used the AIC with samples of less than 100, where the AICc would be much more appropriate. Fifteen analyses involving small samples (less than 100) did not specify the goodness-of-fit method, and thus it is not clear whether the most suitable criterion was used. Only two of the GLMM analyses reviewed used the AICc with small samples (149 subjects).

In general, statistical methods were insufficiently described, and consistent with the findings of Casals et al. (2014) we found that very little information was given about the fixed effects test. When performing likelihood ratio tests of fixed effects it is recommended to use maximum likelihood to estimate parameters (Bolker et al., 2009; Cheng et al., 2010), although this method is not recommended for small and moderate sample sizes (Pinheiro and Bates, 2000). In our review, six GLMM analyses used the likelihood ratio test without specifying the estimation method. Furthermore, in three of the six GLMM analyses that used the likelihood ratio test for fixed effects, the sample included fewer than 100 participants.

Regarding statistical modeling, we did not quantify the strategies for model building (forward selection, backward elimination, best subset, and stepwise procedures) as they were not, as a rule, described. Although a little over half the GLMM analyses did perform statistical modeling, almost all the remaining studies used a full model containing all available predictor variables, reflecting a tendency to keep psychological variables in the model even if they are not significant. Generally speaking, statistical modeling is only advisable if the number of predictor variables is large. Full models give unbiased estimates, but they also contain insignificant predictors. The decision over whether to perform statistical modeling for inference will depend on the study objectives. For inference, stepwise procedures have been criticized because the order of entry or removal of the parameters can influence the selection result, the parameter estimates can be biased, and multiple tests involved in the procedures inflate type I errors (Burnham and Anderson, 2002). Thiele and Markussen (2012) recommend that model building is done by backward model selection, and with GLMM the best subset modeling can easily become computationally expensive when there are multiple fixed or random effects.

Over 90% of the GLMM analyses reviewed did not report the test used for the random effects, which is consistent with findings in the field of clinical medicine (Casals et al., 2014). In general, the likelihood ratio test is appropriate for random effects inferences, but it is only applicable to nested models. This means that information must be provided about the hierarchical structure. The analyses reviewed here most often dealt with a single random effect encompassing variation between participants.

Repeated measures are common in psychology, and GLMMs allow the autocorrelation of such data to be modeled using structured covariance matrices, such as the autoregressive. However, the structure of the covariance matrix was rarely reported in the analyses reviewed here. Consequently, it is unclear whether the tendency in psychology is to use structured or unstructured covariance matrices. It should also be noted that not all statistical software allows the researcher to define the covariance structure when using GLMMs (Thiele and Markussen, 2012).

Regarding overdispersion, and despite the fact that ignoring this phenomenon can cause problems, 90.7% of the GLMM analyses did not specify whether it was assessed. This is an identical result to that obtained by Casals et al. (2014) in the field of medicine. However, it should be noted that overdispersion is only relevant with some distributions.

In addition to all the information related to the use of GLMMs, it is also important to indicate the statistical software used, as the choice can mean differences in estimation methods and computation time. In the analyses reviewed here, the most popular statistical software suites were SAS and R. The glimmix procedure was the first package used to fit GLMMs and it is the standard procedure in SAS. Among other available packages for fitting GLMMs, we found that lme4 (implemented in R) was widely used. Other software suites (e.g., SPSS, STATA, and HLM) were less common. Despite the availability of statistical software, GLMMs are difficult to apply, even for statisticians. Furthermore, it is quite often the case that the models do not converge, especially when there are many random and fixed effects.

Our primary focus in this article has been on the quality of reporting in studies involving GLMMs published in the field of psychology, in other words, whether the information provided is sufficient for researchers with experience of these models to assess the methodological quality of studies and the validity of the results obtained. Consistent with what Casals et al. (2014) observed in clinical medicine, we found that report quality in psychology was poor. A possible explanation for this is that the psychological literature frequently uses models with only fixed effects, even though the use of GLMMs is well known in statistical literature. Because applied researchers in psychology are not, as yet, accustomed to using models in which a lot of information must be taken into account, there is a tendency to report statistical significance without considering other aspects that are important for decision making.

Although our aim in this article was to analyze the reporting of key information when using GLMMs, rather than to detect incongruities in their application, we nonetheless found that they were not always used appropriately. Given that this was evident even with the limited information reported in studies, we suspect that the analyses may well include a high number of model misspecifications. This highlights the need for researchers to conduct these analyses with caution and a good understanding of GLMMs.

After reviewing the publications included in the systematic review, we believe it is important to have minimum standardized guidelines for presenting the results of GLMMs in psychology journals. The minimum information that should be provided, in addition to that required in any statistical analysis (i.e., characteristics of the design, sample, groups, missing data, effect size, *p*-values, etc.), is as follows: measurement scale and distribution of the dependent variable, link function, estimation method, goodness-of-fit method, fixed effects test, statistical modeling, random effects test, variance estimates of random effects, covariance structure for repeated measures, overdispersion evaluation, and software package. Of particular interest is the inclusion of random effects in a GLMM analysis. Stroup et al. (2018) give recommendations and examples on how to configure the random effects structure. Information about power calculation should also be provided and would contribute to improving the quality of GLMM analysis in psychology. For power calculation, readers may consult Stroup (2013) and Stroup et al. (2018).

Overall, our results indicate that there is a need for improved reporting when GLMMs are used in psychology, suggesting that within this field there is a lack of knowledge about what information from a GLMM analysis must be presented. In fact, most of the important information about GLMMs was not stated in the majority of articles included in our systematic review. This lack of reporting about key aspects (e.g., estimation method, link function, goodness-of-fit method, and overdispersion evaluation) makes it difficult to assess the adequacy of the GLMM approaches used. Importantly, not a single article of those reviewed reported all the necessary information.

Although we recognize that the proper use of GLMMs requires a deep understanding of the technique, we hope that this article serves to encourage researchers in psychology to explore and apply these models. Their appropriate application will thus become increasingly common in the field.

## Data Availability Statement

The raw data supporting the conclusions of this article will be made available by the authors, without undue reservation.

## Author Contributions

RB conceived and designed the study, performed the coding, analyzed the data, and drafted and edited the manuscript. MB reviewed the manuscript’s content. RA recorded the characteristics of the journals included in the systematic review. RB and MB were responsible for the reliability of coding (first and second reviewer, respectively). RB, RA, and MB developed the coding manual. All authors contributed to the article and approved the submitted version.

## Conflict of Interest

The authors declare that the research was conducted in the absence of any commercial or financial relationships that could be construed as a potential conflict of interest.

## References

[B1] Aiken L. S. Mistler S. A. Coxe S. West S. G. (2015). Analyzing count variables in individuals and groups: single level and multilevel models. *Group Process Intergroup Relat.* 18 290–314. 10.1177/1368430214556702

[B2] Arnau J. Bendayan R. Blanca M. J. Bono R. (2014). Should we rely on the kenward–roger approximation when using linear mixed models if the groups have different distributions? *Br. J. Math. Stat. Psychol.* 67 408–429. 10.1111/bmsp.12026 24028625

[B3] Atkins D. C. Baldwin S. A. Zheng C. Gallop R. J. Neighbors C. (2013). A tutorial on count regression and zero-altered count models for longitudinal substance use data. *Psychol. Addict. Behav.* 27 166–177. 10.1037/a0029508 22905895 PMC3513584

[B4] Atkins D. C. Gallop R. J. (2007). Rethinking how family researchers model infrequent outcomes: a tutorial on count regression and zero-inflated models. *J. Fam. Psychol.* 21 726–735. 10.1037/0893-3200.21.4.726 18179344

[B5] Bauer D. J. Sterba S. K. (2011). Fitting multilevel models with ordinal outcomes: performance of alternatives specifications and methods of estimation. *Psychol. Methods* 16 373–390. 10.1037/a0025813 22040372 PMC3252624

[B6] Bell M. L. Grunwald G. K. (2011). Small sample estimation properties of longitudinal count models. *J. Stat. Comput. Simul.* 81 1067–1079. 10.1080/00949651003674144

[B7] Blanca M. J. Alarcón R. Arnau J. Bono R. Bendayan R. (2017). Non-normal data: is ANOVA still a valid option? *Psicothema* 29 552–557. 10.7334/psicothema2016.383 29048317

[B8] Blanca M. J. Alarcón R. Bono R. (2018). Current practices in data analysis procedures in psychology: what has changed? *Front. Psychol.* 9:2558. 10.3389/fpsyg.2018.02558 30618979 PMC6300498

[B9] Blanca M. J. Arnau J. López-Montiel D. Bono R. Bendayan R. (2013). Skewness and kurtosis in real data samples. *Methodology* 9 78–84. 10.1027/1614-2241/a000057

[B10] Bolker B. M. Brooks M. E. Clark C. J. Geange S. W. Poulsen J. R. Setevens M. H. (2009). Generalized linear mixed models: a practical guide for ecology and evolution. *Trends Ecol. Evol.* 24 127–135. 10.1016/j.tree.2008.10.008 19185386

[B11] Bono R. Arnau J. Alarcón R. Blanca M. J. (2020). Bias, precision, and accuracy of skewness and kurtosis estimators for frequently used continuous distributions. *Symmetry* 12:19. 10.3390/sym12010019

[B12] Bono R. Arnau J. Vallejo G. (2008). Analysis techniques applied to longitudinal data in psychology and health sciences in the period 1985-2005. *Papeles Psicólogo* 29 136–146.

[B13] Bono R. Blanca M. J. Arnau J. Gómez-Benito J. (2017). Non-normal distributions commonly used in health, education, and social sciences: a systematic review. *Front. Psychol.* 8:1602. 10.3389/fpsyg.2017.01602 28959227 PMC5603665

[B14] Breslow N. E. Clayton D. G. (1993). Approximate inference in generalized linear mixed models. *J. Am. Stat. Assoc.* 88 9–25. 10.1080/01621459.1993.10594284

[B15] Brown H. Prescott R. (2006). *Applied Mixed Models in Medicine*, 2nd Edn. Hoboken, NJ: John Wiley & Sons.

[B16] Burnham K. P. Anderson D. R. (2002). *Model Selection and Multimodel Inference: A Practical Information-Theoretic Approach*, 2nd Edn. New York, NY: Springer.

[B17] Casals M. Girabent-Farrés M. Carrasco J. L. (2014). Methodological quality and reporting of generalized linear mixed models in clinical medicine (2000–2012): a systematic review. *PLoS One* 9:e112653. 10.1371/journal.pone.0112653 25405342 PMC4236119

[B18] Charnigo R. Kryscio R. Bardo M. T. Lynam D. Zimmerman R. S. (2011). Joint modeling of longitudinal data in multiple behavioral change. *Eval. Health Prof.* 34 181–200. 10.1177/0163278710392982 21196429 PMC3092819

[B19] Cheng J. Edwards L. J. Maldonado-Molina M. M. Komro K. A. Muller K. E. (2010). Real longitudinal data analysis for real people: building a good enough mixed model. *Stat. Med.* 29 504–520. 10.1002/sim.3775 20013937 PMC2811235

[B20] Cnnan A. Laird N. M. Slasor P. (1998). Tutorial in biostatistics: using the general linear mixed model to analyse unbalanced repeated measures and longitudinal data. *Stat. Med.* 16 2349–2380. 10.1002/(sici)1097-0258(19971030)16:20<2349::aid-sim667>3.0.co;2-e 9351170

[B21] Cohen J. (1968). Weighted kappa: nominal scale agreement provision for scaled disagreement or partial credit. *Psychol. Bull.* 70 213–220. 10.1037/h0026256 19673146

[B22] Counsell A. Harlow L. L. (2017). Reporting practices and use of quantitative methods in Canadian journal articles in psychology. *Can. Psychol.* 58 140–147. 10.1037/cap0000074 28684887 PMC5494980

[B23] Coupé C. (2018). Modeling linguistic variables with regression models: addressing non-Gaussian distributions, non-independent observations, and non-linear predictors with random effects and generalized additive models for location, scale, and shape. *Front. Psychol.* 9:513. 10.3389/fpsyg.2018.00513 29713298 PMC5911484

[B24] Coxe S. West S. G. Aiken L. S. (2009). The analysis of count data: a gentle introduction to Poisson regression and its alternatives. *J. Pers. Assess.* 91 121–136. 10.1080/00223890802634175 19205933

[B25] Dean C. B. Nielsen J. (2007). Generalized linear mixed models: a review and some extensions. *Lifetime Data Anal.* 13 497–512. 10.1007/s10985-007-9065-x 18000755

[B26] Der G. Everitt B. (2006). *Statistical Analysis of Medical Data Using SAS.* London: Chapman & Hall.

[B27] Edgington E. S. (1964). A tabulation of inferential statistics used in psychology journals. *Am. Psychol.* 19 202–203. 10.1037/h0039177

[B28] Edgington E. S. (1974). A new tabulation of statistical procedures used in APA journals. *Am. Psychol.* 29 25–26. 10.1037/h0035846

[B29] Elosua P. De Boeck P. (2020). Educational assessment issues in linguistically diverse contexts: a case study using a generalized linear mixed model. *Lang. Cult. Curric.* 33 305–318. 10.1080/07908318.2019.1662432

[B30] Ferreira E. B. Rocha M. C. Mequelino D. B. (2012). Monte carlo evaluation of the ANOVA’s F and Kruskal-wallis tests under binomial distribution. *Sigmae* 1 126–139.

[B31] Garson G. D. (2013). “Fundamentals of hierarchical linear and multilevel modeling,” in *Hierarchical Linear Modeling: Guide and Applications*, ed. Garson G. D. (Thousand Oaks, CA: Sage), 3–25. 10.4135/9781483384450.n1

[B32] Heck R. H. Thomas S. L. Tabata L. N. (2010). *Multilevel and Longitudinal Modeling with IBM SPSS.* Abingdon: Routledge.

[B33] Heck R. H. Thomas S. L. Tabata L. N. (2012). *Multilevel Modeling of Categorical Outcomes Using IBM SPSS.* Abingdon: Routledge.

[B34] Hilbe J. M. (2011). *Negative Binomial Regression*, 2nd Edn. Cambridge: Cambridge University Press.

[B35] Hox J. (2002). *Multilevel Analysis: Techniques and Applications.* Mahwah, NJ: Lawrence Erlbaum Associates.

[B36] Jiang J. (2007). *Linear and Generalized Linear Mixed Models and Their Applications.* New York, NY: Springer.

[B37] Johnson P. C. D. Barry S. J. E. Ferguson H. M. Müller P. (2015). Power analysis for generalized linear mixed models in ecology and evolution. *Methods Ecol. Evol.* 6 133–142. 10.1111/2041-210X.12306 25893088 PMC4394709

[B38] Kain M. P. Bolker B. M. McCoy M. W. (2015). A practical guide and power analysis for GLMMs: detecting among treatment variation in random effects. *PeerJ* 3:e1226. 10.7717/peerj.1226 26401446 PMC4579019

[B39] Kanji G. K. (1976). Effect of non-normality on the power in analysis of variance: a simulation study. *Int. J. Math. Educ. Sci. Technol.* 7 155–160. 10.1080/0020739760070204

[B40] Kauermann G. Ormerod J. T. Wand M. P. (2010). Parsimonious classification via generalized linear mixed models. *J. Classif.* 27 89–110. 10.1007/s00357-010-9045-9

[B41] Keselman H. J. Algina J. Kowalchuk R. K. Wolfinger R. D. (1998). A comparison of two approaches for selecting covariance structures in the analysis of repeated measures. *Commun. Stat. Simul. Comput.* 27 591–604. 10.1080/03610919808813497

[B42] Khan A. Rayner G. D. (2003). Robustness to non-normality of common tests for the many-sample location problem. *J. Appl. Math. Decis. Sci.* 7 187–206. 10.1155/S1173912603000178

[B43] Kieffer K. M. Reese R. J. Thompson B. (2001). Statistical techniques employed in AERJ and JCP articles from 1988 to 1997: a methodological review. *J. Exp. Educ.* 69 280–309. 10.1080/00220970109599489

[B44] Landis J. Koch G. (1977). The measurement of observer agreement for categorical data. *Biometrics* 33 159–174. 10.2307/2529310 843571

[B45] Lei M. Lomax R. G. (2005). The effect of varying degrees on nonnormality in structural equation modeling. *Struct. Equ. Model.* 12 1–27. 10.1207/s15328007sem1201_1

[B46] McNeish D. (2016). Estimation methods for mixed logistic models with few clusters. *Multivar. Behav. Res.* 51 790–804. 10.1080/00273171.2016.1236237 27802068

[B47] Micceri T. (1989). The unicorn, the normal curve, and other improbable creatures. *Psychol. Bull.* 105 156–166. 10.1037/0033-2909.105.1.156

[B48] Milanzi E. Alonso A. Molenberghs G. (2012). Ignoring overdispersion in hierarchical loglinear models: possible problems and solutions. *Stat. Med.* 31 1475–1482. 10.1002/sim.4482 22362329

[B49] Moher D. Liberati A. Tetzlaff J. Altman D. G. (2009). Preferred reporting items for systematic reviews and meta-analyses: the PRISMA statement. *PLoS Med.* 6:e1000097. 10.1371/journal.pmed.1000097 19621072 PMC2707599

[B50] Moscatelli A. Mezzetti M. Lacquaniti F. (2012). Modeling psychophysical data at the population-level: the generalized linear mixed model. *Journal of Vision* 12 1–17. 10.1167/12.11.26 23104819

[B51] Pan J. Thompson R. (2003). Gauss-Hermite quadrature approximation for estimation in generalized linear mixed models. *Comput. Stat.* 18 57–78. 10.1007/s001800300132

[B52] Pinheiro J. C. Bates D. M. (2000). *Mixed-Effects Models in S and SPLUS.* New York, NY: Springer-Verlag.

[B53] Platt R. W. Leroux B. G. Breslow N. (1999). Generalized linear mixed models for meta-analysis. *Stat. Med.* 18 643–654. 10204195 10.1002/(sici)1097-0258(19990330)18:6<643::aid-sim76>3.0.co;2-m

[B54] Reis H. T. Stiller J. (1992). Publication trends in JPSP: a three-decade review. *Pers. Soc. Psychol.Bull.* 18 465–472. 10.1177/0146167292184011

[B55] Schinka J. A. LaLone L. Broeckel J. A. (1997). Statistical methods in personality assessment research. *J. Pers. Assess.* 68 487–496. 10.1207/s15327752jpa6803_2 16372864

[B56] Schmider E. Ziegler M. Danay E. Beyer L. Bühner M. (2010). Is it really robust? Reinvestigating the robustness of ANOVA against violations of the normal distribution assumption. *Methodology* 6 147–151. 10.1027/1614-2241/a000016

[B57] Skidmore S. T. Thompson B. (2010). Statistical techniques used in published articles: a historical review of reviews. *Educ. Psychol. Meas.* 70 777–795. 10.1177/0013164410379320

[B58] Skrondal A. Rabe-Hesketh S. (2003). Some applications of generalized linear latent and mixed models in epidemiology: repeated measures, measurement error and multilevel modeling. *Norsk Epidemiol.* 13 265–278. 10.5324/nje.v13i2.295

[B59] Stroup W. W. (2013). *Generalized Linear Mixed Models: Modern Concepts, Methods and Applications.* Abingdon: Taylor and Francis.

[B60] Stroup W. W. Milliken G. A. Claassen E. A. Wolfinger R. D. (2018). *SAS for Mixed Models: Introduction and Basic Applications.* Cary, NC: SAS Institute Inc.

[B61] Tango T. (2017). *Repeated Measures Design with Generalized Linear Mixed Models for Randomized Controlled Trials.* London: Chapman & Hall.

[B62] Thiele J. Markussen B. (2012). Potential of GLMM in modelling invasive spread. *CAB Reviews* 7 1–10. 10.1079/PAVSNNR20127016

[B63] Vehtari A. Gelman A. Gabry J. (2017). Practical Bayesian model evaluation using leave-one-out cross-validation and WAIC. *Stat. Comput.* 27 1413–1432. 10.1007/s11222-016-9696-4

[B64] Watanabe S. (2010). Asymptotic equivalence of Bayes cross-validation and widely applicable information criterion in singular learning theory. *J. Mach. Learn. Res.* 11 3571–3594.

