# Supplementary material for: Report Quality of Generalized Linear Mixed Models in Psychology: A Systematic Review
Source: Front Psychol. 2021 Apr 22;12:666182. doi: 10.3389/fpsyg.2021.666182 (PMC8100208; doi:10.3389/fpsyg.2021.666182)
Supplement: Supplementary file 2 [file Data_Sheet_2.docx]

Appendix 2. Number of articles using GLMMs and published during the period 2014-2018 in a total of 198 journals.

| Journal | N |
| --- | --- |
| ACADEMIC PEDIATRICS  ACCIDENT ANALYSIS AND PREVENTION  ACTA NEUROLOGICA SCANDINAVICA  ADDICTION  ADDICTIVE BEHAVIORS  AGING & MENTAL HEALTH  AGING NEUROPSYCHOLOGY AND COGNITION  AIDS AND BEHAVIOR  AIDS CARE-PSYCHOLOGICAL AND SOCIO-MEDICAL ASPECTS OF AIDS/HIV  ALCOHOL  ALCOHOLISM-CLINICAL AND EXPERIMENTAL RESEARCH  ALZHEIMERS & DEMENTIA  AMERICAN JOURNAL OF COMMUNITY PSYCHOLOGY  AMERICAN JOURNAL OF DRUG AND ALCOHOL ABUSE  AMERICAN JOURNAL OF HEALTH BEHAVIOR  AMERICAN JOURNAL OF HEALTH PROMOTION  AMERICAN JOURNAL OF PHYSICAL ANTHROPOLOGY  AMERICAN JOURNAL OF PHYSICAL MEDICINE & REHABILITATION  AMERICAN JOURNAL OF PREVENTIVE MEDICINE  AMERICAN JOURNAL OF PRIMATOLOGY  AMERICAN JOURNAL OF PSYCHIATRY  AMERICAN JOURNAL OF PUBLIC HEALTH  ANNALS OF BEHAVIORAL MEDICINE  ANNALS OF EPIDEMIOLOGY  ANNALS OF FAMILY MEDICINE  ANTHROZOOS  APPLIED ANIMAL BEHAVIOUR SCIENCE  APPLIED PSYCHOLOGICAL MEASUREMENT  ARCHIVES OF PHYSICAL MEDICINE AND REHABILITATION  ARCHIVES OF PSYCHIATRIC NURSING  ARCHIVES OF SEXUAL BEHAVIOR  AUSTRALIAN AND NEW ZEALAND JOURNAL OF PUBLIC HEALTH  BEHAVIORAL NEUROSCIENCE  BIOLOGICAL PSYCHOLOGY  BIPOLAR DISORDERS  BMC PSYCHIATRY  BMC PUBLIC HEALTH  BMJ OPEN  BRAIN  BRAIN STIMULATION  BRITISH JOURNAL OF EDUCATIONAL PSYCHOLOGY  BRITISH JOURNAL OF MATHEMATICAL & STATISTICAL PSYCHOLOGY  BRITISH JOURNAL OF PSYCHIATRY  CANADIAN JOURNAL OF PSYCHIATRY-REVUE CANADIENNE DE PSYCHIATRIE  CANADIAN MEDICAL ASSOCIATION JOURNAL  CBE-LIFE SCIENCES EDUCATION  CHILD ABUSE & NEGLECT  CHRONOBIOLOGY INTERNATIONAL  CIN-COMPUTERS INFORMATICS NURSING  COGNITIVE THERAPY AND RESEARCH  COMPREHENSIVE PSYCHIATRY  COMPUTERS IN HUMAN BEHAVIOR  CONSCIOUSNESS AND COGNITION  CONTEMPORARY CLINICAL TRIALS  DEMENTIA-INTERNATIONAL JOURNAL OF SOCIAL RESEARCH AND PRACTICE  DEVELOPMENTAL PSYCHOLOGY  DIABETES CARE  DRUG AND ALCOHOL DEPENDENCE  DRUG AND ALCOHOL REVIEW  EARLY CHILDHOOD RESEARCH QUARTERLY  EDUCATIONAL AND PSYCHOLOGICAL MEASUREMENT  ENVIRONMENTAL HEALTH PERSPECTIVES  EUROPEAN JOURNAL OF PERSONALITY  EUROPEAN PSYCHIATRY  EXPERIMENTAL BRAIN RESEARCH  FRONTIERS IN HUMAN NEUROSCIENCE  FRONTIERS IN PSYCHIATRY  FRONTIERS IN PSYCHOLOGY  GERIATRICS & GERONTOLOGY INTERNATIONAL  HEADACHE  HEALTH & PLACE  HEALTH EDUCATION & BEHAVIOR  HEALTH PSYCHOLOGY  HIPPOCAMPUS  HUMAN MOVEMENT SCIENCE  HUMAN RESOURCE DEVELOPMENT QUARTERLY  INTERNATIONAL ARCHIVES OF OCCUPATIONAL AND ENVIRONMENTAL HEALTH  INTERNATIONAL JOURNAL FOR EQUITY IN HEALTH  INTERNATIONAL JOURNAL FOR QUALITY IN HEALTH CARE  INTERNATIONAL JOURNAL OF BEHAVIORAL NUTRITION AND PHYSICAL ACTIVITY  INTERNATIONAL JOURNAL OF DRUG POLICY  INTERNATIONAL JOURNAL OF EATING DISORDERS  INTERNATIONAL JOURNAL OF ENVIRONMENTAL RESEARCH AND PUBLIC HEALTH  INTERNATIONAL JOURNAL OF GERIATRIC PSYCHIATRY  INTERNATIONAL JOURNAL OF GYNECOLOGY & OBSTETRICS  INTERNATIONAL JOURNAL OF INTERCULTURAL RELATIONS  INTERNATIONAL JOURNAL OF LANGUAGE & COMMUNICATION DISORDERS  INTERNATIONAL JOURNAL OF MEDICAL INFORMATICS  INTERNATIONAL JOURNAL OF NURSING STUDIES  INTERNATIONAL JOURNAL OF PUBLIC HEALTH  INTERNATIONAL JOURNAL OF SPORTS PHYSIOLOGY AND PERFORMANCE  INTERNATIONAL JOURNAL OF STD & AIDS  INTERNATIONAL JOURNAL OF TUBERCULOSIS AND LUNG DISEASE  JAMA PSYCHIATRY  JOURNAL OF ADDICTION MEDICINE  JOURNAL OF ADOLESCENCE  JOURNAL OF ADOLESCENT HEALTH  JOURNAL OF ADVANCED NURSING  JOURNAL OF AFFECTIVE DISORDERS  JOURNAL OF AMERICAN COLLEGE HEALTH  JOURNAL OF ANXIETY DISORDERS  JOURNAL OF BEHAVIORAL MEDICINE  JOURNAL OF CANCER SURVIVORSHIP  JOURNAL OF CHILD AND FAMILY STUDIES  JOURNAL OF CLINICAL PSYCHIATRY  JOURNAL OF CLINICAL PSYCHOLOGY IN MEDICAL SETTINGS  JOURNAL OF COMMUNITY HEALTH  JOURNAL OF COMPARATIVE PSYCHOLOGY  JOURNAL OF CONSULTING AND CLINICAL PSYCHOLOGY  JOURNAL OF DEVELOPMENTAL AND BEHAVIORAL PEDIATRICS  JOURNAL OF EDUCATIONAL MEASUREMENT  JOURNAL OF ENVIRONMENTAL MANAGEMENT  JOURNAL OF EPIDEMIOLOGY AND COMMUNITY HEALTH  JOURNAL OF ETHNICITY IN SUBSTANCE ABUSE  JOURNAL OF EXPERIMENTAL PSYCHOPATHOLOGY  JOURNAL OF FAMILY VIOLENCE  JOURNAL OF GENERAL INTERNAL MEDICINE  JOURNAL OF HEAD TRAUMA REHABILITATION  JOURNAL OF MANAGEMENT  JOURNAL OF OCCUPATIONAL AND ENVIRONMENTAL MEDICINE  JOURNAL OF PAIN AND SYMPTOM MANAGEMENT  JOURNAL OF PHYSICAL ACTIVITY & HEALTH  JOURNAL OF PSYCHIATRIC RESEARCH  JOURNAL OF PSYCHOLOGY IN AFRICA  JOURNAL OF PSYCHOSOMATIC RESEARCH  JOURNAL OF RURAL HEALTH  JOURNAL OF SCHOOL HEALTH  JOURNAL OF SCIENCE AND MEDICINE IN SPORT  JOURNAL OF SPEECH LANGUAGE AND HEARING RESEARCH  JOURNAL OF SPORTS SCIENCES  JOURNAL OF STUDIES ON ALCOHOL AND DRUGS  JOURNAL OF SUBSTANCE ABUSE TREATMENT  JOURNAL OF THE ACADEMY OF NUTRITION AND DIETETICS  JOURNAL OF THE AMERICAN ACADEMY OF CHILD AND ADOLESCENT PSYCHIATRY  JOURNAL OF THE AMERICAN MEDICAL DIRECTORS ASSOCIATION  JOURNAL OF THE AMERICAN MEDICAL INFORMATICS ASSOCIATION  JOURNAL OF TRAUMATIC STRESS  JOURNAL OF VISION  JOURNALS OF GERONTOLOGY SERIES A-BIOLOGICAL SCIENCES AND MEDICAL SCIENCES  JOVE-JOURNAL OF VISUALIZED EXPERIMENTS  LANCET PSYCHIATRY  LATERALITY  LEARNING AND INDIVIDUAL DIFFERENCES  MEDICINE  METABOLIC BRAIN DISEASE  METACOGNITION AND LEARNING  MILITARY MEDICINE  MINDFULNESS  MULTIVARIATE BEHAVIORAL RESEARCH  NEUROIMAGE  NICOTINE & TOBACCO RESEARCH  NUTRITION JOURNAL  OBESITY  PAIN  PARKINSONISM & RELATED DISORDERS  PATIENT EDUCATION AND COUNSELING  PERCEPTUAL AND MOTOR SKILLS  PERSONALITY AND INDIVIDUAL DIFFERENCES  PLOS ONE  PREHOSPITAL AND DISASTER MEDICINE  PREVENTING CHRONIC DISEASE  PREVENTION SCIENCE  PREVENTIVE MEDICINE  PROCEEDINGS OF THE ROYAL SOCIETY B-BIOLOGICAL SCIENCES  PSYCHIATRY AND CLINICAL NEUROSCIENCES  PSYCHO-ONCOLOGY  PSYCHOLOGY AND AGING  PSYCHOLOGY IN THE SCHOOLS  PSYCHOLOGY OF ADDICTIVE BEHAVIORS  PSYCHONEUROENDOCRINOLOGY  PSYCHOPATHOLOGY  PSYCHOSOMATIC MEDICINE  PUBLIC HEALTH  QUALITY OF LIFE RESEARCH  QUARTERLY JOURNAL OF EXPERIMENTAL PSYCHOLOGY  REPRODUCTIVE HEALTH  RESTORATIVE NEUROLOGY AND NEUROSCIENCE  SCANDINAVIAN JOURNAL OF PSYCHOLOGY  SCANDINAVIAN JOURNAL OF PUBLIC HEALTH  SCHIZOPHRENIA BULLETIN  SCHIZOPHRENIA RESEARCH  SCHOOL MENTAL HEALTH  SCHOOL PSYCHOLOGY QUARTERLY  SCIENTIFIC REPORTS  SEXUALLY TRANSMITTED DISEASES  SEXUALLY TRANSMITTED INFECTIONS  SLEEP AND BREATHING  SOCIAL PSYCHIATRY AND PSYCHIATRIC EPIDEMIOLOGY  SOCIAL SCIENCE & MEDICINE  STUDIES IN FAMILY PLANNING  SUBSTANCE ABUSE  SUBSTANCE USE & MISUSE  SUICIDE AND LIFE-THREATENING BEHAVIOR  SUPPORTIVE CARE IN CANCER  TRANSLATIONAL BEHAVIORAL MEDICINE  TRANSPORTATION RESEARCH PART F-TRAFFIC PSYCHOLOGY AND BEHAVIOUR  TRIALS  ZOO BIOLOGY | 1  5  1  4  5  1  1  4  1  1  2  1  1  1  3  6  2  1  1  2  1  1  2  4  1  1  1  1  1  1  2  1  1  1  1  3  6  9  1  1  2  1  1  1  1  1  1  1  1  1  1  2  3  2  1  1  1  5  2  1  2  1  1  2  2  2  1  3  2  1  2  2  2  1  3  1  1  1  1  2  1  2  1  2  1  1  1  1  1  1  1  1  1  1  1  1  5  1  2  1  1  1  1  2  3  1  1  1  1  2  1  1  1  1  1  1  1  1  1  1  1  1  3  1  1  1  3  1  1  1  2  2  1  2  2  2  1  1  1  1  2  1  2  2  1  2  1  1  1  1  3  1  1  1  1  1  1  1  18  1  1  2  1  1  1  1  1  1  1  1  1  1  1  3  1  1  1  1  1  1  2  1  2  1  1  2  1  1  2  1  1  2  1  1  1  1  1  1 |
| N Total | 316 |
