# Supplementary material for: Report Quality of Generalized Linear Mixed Models in Psychology: A Systematic Review
Source: Front Psychol. 2021 Apr 22;12:666182. doi: 10.3389/fpsyg.2021.666182 (PMC8100208; doi:10.3389/fpsyg.2021.666182)
Supplement: Supplementary file 3 [file Data_Sheet_3.docx]

Appendix 3. Number of articles using GLMMs and published during the period 2014-2018, by JCR category.

| JCR category | N |
| --- | --- |
| SUBSTANCE ABUSE  GERONTOLOGY  SOCIAL SCIENCES, BIOMEDICAL  PUBLIC, ENVIRONMENTAL AND OCCUPATIONAL HEALTH  EVOLUTIONARY BIOLOGY  ZOOLOGY  VETERINARY SCIENCES  SOCIAL SCIENCE, INTERDISCIPLINARY  PEDIATRICS  ERGONOMICS  CLINICAL NEUROLOGY  PSYCHOLOGY, DEVELOPMENTAL  SOCIAL WORK  REHABILITATION  PSYCHIATRY  PSYCHOLOGY, MULTIDISCIPLINARY  PRIMARY HEALTH CARE  AGRICULTURE, DAIRY AND ANIMAL HEALTH  PSYCHOLOGY, MATHEMATICAL  NURSING  BEHAVIORAL SCIENCES  MEDICINE, GENERAL AND INTERNAL  PSYCHOLOGY, EDUCATIONAL  STATISTICS AND PROBABILITY  EDUCATION, SCIENTIFIC DISCIPLINES  BIOLOGY  PSYCHOLOGY, CLINICAL  PSYCHOLOGY, EXPERIMENTAL  MEDICINE, RESEARCH AND EXPERIMENTAL  ENDOCRINOLOGY AND METABOLISM  TOXICOLOGY  PSYCHOLOGY, SOCIAL  NEUROSCIENCES  PSYCHOLOGY  GERIATRICS AND GERONTOLOGY  HEALTH POLICY AND SERVICES  SOCIAL SCIENCES, MATHEMATICAL METHODS  SPORT SCIENCES  INDUSTRIAL RELATIONS AND LABOR  NUTRITION AND DIETETICS  OBSTETRICS AND GYNECOLOGY  LINGUISTICS  HEALTH CARE SCIENCES AND SERVICES  INFECTIOUS DISEASES  RESPIRATORY SYSTEM  FAMILY STUDIES  ENVIRONMENTAL SCIENCES  PSYCHOLOGY, APPLIED  OPHTHALMOLOGY  MULTIDISCIPLINARY SCIENCES  NEUROIMAGING  ANESTHESIOLOGY  EMERGENCY MEDICINE  DEMOGRAPHY  ANTHROPOLOGY  EDUCATION & EDUCATIONAL RESEARCH  PSYCHOLOGY, BIOLOGICAL  PHYSIOLOGY  MEDICAL INFORMATICS  SOCIAL ISSUES | 33  8  8  43  1  3  2  4  7  5  7  7  2  4  27  10  1  1  1  4  1  14  12  1  1  1  14  6  3  2  1  2  4  5  2  4  2  6  1  4  1  2  2  4  1  3  1  2  1  20  1  1  1  1  2  3  1  1  3  1 |
| N Total | 316 |
